# Proton pump inhibitors use and risk of type 2 diabetes mellitus: correlation analysis, prediction model construction, and key genes identification

**DOI:** 10.3389/fphar.2025.1580090

**Published:** 2025-04-29

**Authors:** Cuilv Liang, Yin Zhang

**Affiliations:** Department of Pharmacy, The Second Affiliated Hospital of Fujian Medical University, Quanzhou, China

**Keywords:** proton pump inhibitors, type 2 diabetes mellitus, correlation analysis, prediction model, gene identification

## Abstract

**Introduction:**

Prior cohort studies reported paradoxical results between proton pump inhibitor (PPI) usage and the risk of type 2 diabetes mellitus (T2DM). We investigated the correlation between the use of PPIs and T2DM risk, constructed predictive models, and identified the key genes involved.

**Methods:**

In the correlation analysis, we extracted and analyzed the data from the National Health and Nutrition Examination Survey (NHANES) database and the FDA Adverse Event Reporting System (FAERS) database to examine the relationship between the use of PPIs and T2DM risk. Then, a nomogram was constructed to estimate the T2DM risk probability in patients treated with PPIs by using the optimal predictors identified by the least absolute shrinkage and selection operator and logistic regression methods. Finally, we investigated the key genes modulated by PPI usage in patients with T2DM by combining various bioinformatics techniques such as network pharmacology, difference analysis, and weighted gene co-expression network analysis.

**Results:**

In the NHANES database, regardless of whether PPI usage was merely included or used to adjust for covariates, the binomial regression models indicated a positive correlation between PPI usage and T2DM risk (all p < 0.001). In the FAERS database, the T2DM signal for patients using PPIs was significant (lower limit of the reporting odds ratio was greater than 1). Sex, race, age, educational level, obesity, hypertension, and high cholesterol were included in the nomogram to predict the probability of PPI usage-induced T2DM risk (all p < 0.05). By intersecting the key cluster and the intersection of PPI usage-related genes and T2DM-related genes, we finally identified two crucial genes, AGT and JAK2, that may be involved in PPI usage-induced T2DM risk.

**Discussion:**

Our findings revealed that PPI treatment can increase the risk of T2DM. Additionally, we were successful in constructing a new nomogram to identify individuals at high risk of developing T2DM among patients using PPIs and completed a preliminary exploration of possible gene targets and mechanisms. Our study will be useful in alerting clinicians to the T2DM risk involved in PPI treatment and allowing them to take early prevention and intervention measures.

## 1 Introduction

Proton pump inhibitors (PPIs) directly block the final common pathway of gastric acid secretion and are the most effective inhibitors of gastric acid secretion at present. Since the introduction of omeprazole in 1989, PPIs have gradually become the primary drugs for the treatment of acid-related disorders, with the virtues of consistent tolerance, excellent safety, and a stronger ability to inhibit gastric acid (Strand et al., 2017). Several PPIs have been developed, including omeprazole, pantoprazole, lansoprazole, rabeprazole, esomeprazole, and dexlansoprazole, all of which have been approved by several countries. Despite a consistently favorable safety profile of PPIs over the past 3 decades of clinical practice, some concerns have arisen about their adverse effects as a consequence of their gradually universal popularity (Vaezi et al., 2017; Elias and Targownik, 2019).

Diabetes is estimated to affect approximately 530 million adults worldwide, with a global prevalence of 10.5% among adults aged 20–79 years. Total diabetes prevalence primarily reflects type 2 diabetes mellitus (T2DM), which accounts for 96.0% of diabetes cases (GBD 2021 Diabetes Collaborators, 2023. T2DM is a substantial public health issue worldwide. The pursuit of a link between PPI usage and T2DM risk has yielded contradictory results. A prospective analysis of 204,689 participants found that regular use of PPIs was associated with a higher risk of T2DM and the risk increased with longer duration of use (Yuan et al., 2021). However, another retrospective cohort study with a follow-up period of 5 years demonstrated a decreased risk of diabetes in upper gastrointestinal disease patients who used PPIs (Lin et al., 2016). Thus, the correlation between PPI usage and T2DM risk remains unconfirmed.

The National Health and Nutrition Examination Survey (NHANES) was conducted by the Centers for Disease Control and Prevention, United States to gather health and nutrition information from American households (Hartwell et al., 2019). The FDA Adverse Events Reporting System (FAERS) database is a spontaneous reporting system for adverse event (AE) reports arising from the use of marketed drugs in large populations, which can unearth overlooked or rare AEs in clinical studies (Inácio et al., 2017). These two databases are able to utilize large-volume datasets to examine the relationship between individual drugs and individual diseases, and are powerful tools for detecting adverse drug reactions (Li et al., 2024; Liang et al., 2024). Therefore, we employed data from the NHANES and FAERS databases to explore the correlation between PPI usage and T2DM risk. Meanwhile, many researchers have used the NHANES database to build various nomogram prediction models and to achieve positive results (Mao et al., 2024; Wu C. et al., 2024; Wu W. et al., 2024). After exploring the correlation between PPI usage and T2DM risk, we used the NHANES database to construct a predictive model of PPI usage-induced T2DM risk. Bioinformatics technology is an effective approach for exploring the potential mechanisms and key genes associated with the development of disease and the effects of drugs (Xu et al., 2023). In our study, we aimed to investigate the key genes involved in PPI usage-induced T2DM risk by employing various bioinformatics techniques such as network pharmacology, difference analysis, and weighted gene co-expression network analysis (WGCNA). By exploring the correlation between PPI usage and T2DM risk, predictive model construction, and key genes identification, that is helpful for clinicians to be alerted of the risk of T2DM caused by PPI usage, and take early prevention and intervention measures.

## 2 Materials and methods

### 2.1 Sample population in the NHANES database

**FIGURE 1:**
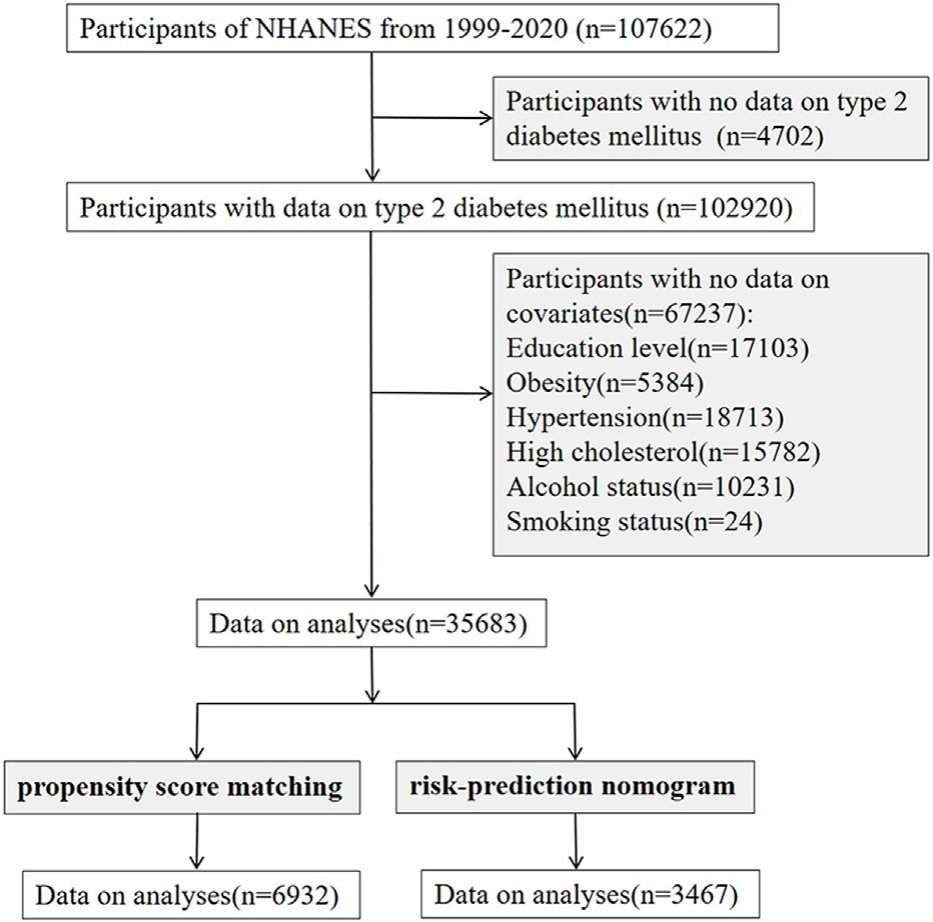
Flowchart of subject selection in NHANES. NHANES, The National Health and Nutrition Examination Survey.

The 1999–2017 dataset from the NHANES database was used in our study. After excluding participants with missing information on T2DM and covariates, we enrolled 35,683 participants. After re-matching by propensity score matching (PSM), we re-studied 6,932 patients (Figure 1). We used the extracted data to investigate the relationship between PPI usage and T2DM in the following three models:1) incorporating PPI usage alone; 2) adjusted for diseases (hypertension, high cholesterol, obesity) that affect T2DM (Wang et al., 2021; Jayedi et al., 2022); 3) adjusted for all covariates that are risk factors for T2DM (Wang et al., 2021; Jayedi et al., 2022; Menke et al., 2015; Spijkerman et al., 2014). Additionally, to discern the effect of different PPI usage on T2DM, we perform subgroup analyses by omeprazole/esomeprazole/pantoprazole (n > 500 patients).

### 2.2 Primary variable information in the NHANES database

The exposure variable of this study was the usage of PPIs. Data on PPI use was gathered through the Prescription Medication Section of the Sample Person Questionnaire. Six PPIs (omeprazole, pantoprazole, lansoprazole, rabeprazole, esomeprazole, and dexlansoprazole) and 3,466 persons who used PPIs were included in the final sample based on information gathered from in-person interviews during the eleven NHANES cycles. We defined T2DM as any of the following (American Diabetes Association Primary Care Advisory Group, 2024): (1) HbA1c levels ≥6.5%; (2) fasting glucose levels ≥126 mg/dL; (3) serum glucose levels exceeding 200 mg/dL as measured by the oral glucose tolerance test; (4) participants who answered YES to the question, “Ever told you had diabetes?” in the self-reported questionnaire.

### 2.3 Covariates information in the NHANES database

The covariates included age, sex (man/woman), race (Mexican and Hispanic American, non-Hispanic white, non-Hispanic black and other), educational level, hypertension, high cholesterol, obesity (body mass index ≥30 kg/m^2^), alcohol status (≥12 times in the past 12 months), and smoking status. The categories for educational level included less than 12th grade/12th grade without a diploma, high school graduate/GED or equivalent, some college or AA degree, and college graduate or above. Participants were classified as having hypertension and high cholesterol if they answered “YES” to the question, “Did your doctor tell you that you have high blood pressure or high cholesterol levels?” Three groups of smoking status were identified: never (less than 100 cigarettes smoked in their lifetime), former (more than 100 cigarettes smoked in their lifetime and not at all presently), and current (more than 100 cigarettes smoked in their lifetime and smoking sometimes or daily).

### 2.4 FAERS database

To mine the FAERS data from 2004 Quarter-1 to 2024 Quarter-2, an updated online web-based analysis tool termed AERSMine (https://research.cchmc.org/aers) was developed (Sarangdhar et al., 2016). We used AERSMine to extract the AEs for PPIs, omeprazole, pantoprazole, lansoprazole, rabeprazole, esomeprazole, and dexlansoprazole. AEs in the FAERS database were scored in accordance with the Medical Dictionary for Regulatory Activities from system organ class, high-level group terms, high-level terms (HLT), preferred terms (PT), and lowest-level terms (Revers et al., 2022). We obtained data for PPIs, omeprazole, pantoprazole, lansoprazole, rabeprazole, esomeprazole, and dexlansoprazole-associated T2DM AEs by searching the FAERS database via the AERSMine tool and employing the search terms of HLT (diabetes mellitus) and PT (T2DM).

### 2.5 Statistical analysis

In the present report, the study population characteristics were expressed as the mean and standard deviation for continuous variables, and categorical variables were presented as percentages. We used three models to assess the association between T2DM and PPI usage in the binomial regression model. Additionally, to exclude the interference of confounding factors, the PSM method was employed with a 1:1 ratio to balance controls and cases, and the data obtained following PSM were also reanalyzed (Kurz et al., 2024).

Next, the extracted data with PPI usage were divided into training and testing groups in a 7: 3 ratio. Based on the data from the training group (n = 2431), we constructed a nomogram to quantify the risk of PPI usage-induced T2DM. To identify the optimal predictive characteristics and reduce the dimensionality of these data, the least absolute shrinkage and selection operator (LASSO) regression estimator was employed. The non-zero coefficients of the LASSO regression model were selectively embraced as potential indicators of PPI-induced T2DM. Following their identification, these possible factors were methodically added to a binomial logistic regression model. Adopting factors with p values <0.05, a nomogram regarding the probability of PPI usage-induced T2DM was created (Zou et al., 2022). Subsequently, data from the testing group (n = 1036) were applied to externally validate the predictive effect of our nomogram.

The accuracy of the risk prediction model was assessed by several metrics, including receiver operating characteristic (ROC) curves, C-index, calibration plots, and decision curve analysis (DCA). The ROC curve area under the curve (AUC) value is close to 1, indicating the robust performance of the prediction model (Harrell et al., 1982). The C-index >0.7 is considered to have good discriminatory power (Longato et al., 2020). In the calibration plot, if the curve lies on the diagonal of the coordinates, it indicates that the predictive power of the model is more accurate (Barda et al., 2020). DCA is applied to assess the clinical utility of nomograms based on the threshold probabilities. The threshold probability can be utilized to yield a net benefit. By graphically analyzing the net benefit and the threshold probability, a decision analysis curve can be derived, which can then be employed to evaluate the net benefit of nomogram-assisted decision making at different threshold probabilities (Van Calster et al., 2018).

Disproportionality analysis is a commonly used method to detect the signal of adverse reactions, which is based on a 2 × 2 table. We applied the proportional reporting ratio (PRR) and the reporting odds ratio (ROR) of disproportionality analysis to determine the signal strength of PPI usage-associated T2DM to guarantee the accuracy of the results (Arai et al., 2023). The 2 × 2 table and formulas for the 2 algorithms are listed in Supplementary Tables S1, S2. When the ROR and PRR lower limit of the 95% confidence interval (CI) was greater than 1 and the number of AE reports was greater than or equal to three, the signal was deemed significant (Yang et al., 2022). The ROR and PRR were calculated in two models: 1) database without restrictions; 2) by excluding patients who already had T2DM, hypertension, hyperlipidemia, and obesity before therapy. IBM SPSS Statistics version 27 (IBM Corp., Armonk, NY), Microsoft Excel version 2013 (Microsoft Corp., Redmond, W), and the “forestplot,” “MASS,” “ggplot2,” “glmnet,” “ISwR,” “caret,” “foreign,” “rms,” “rmda,”, and “reshape2” packages of the R open-source software version 4.2.2 were performed to conduct the statistical analysis.

### 2.6 Identification of crucial targets of T2DM

The raw data of the GSE7014 gene expression dataset used in our analysis were downloaded from the Gene Expression Omnibus (GEO) online database. The following criteria were used to identify differentially expressed genes (DEGs) between T2DM samples and normal controls after raw data normalization: *p* < 0.05 and |fold change| > 1 (Zhang et al., 2023). To display DEGs, heatmap and volcano plots were generated, and the significant genes were labeled. Defined gene sets were identified by using gene set enrichment analysis (GSEA) (Subramanian et al., 2007). GSEA analysis revealed DEG differences between the two biological processes.

By analyzing the GSE7014 gene expression dataset, a gene co-expression network was built using WGCNA (Chen et al., 2022). In order to deal with outlier samples, a hierarchical clustering tree was first generated. Gene correlation matrices and topological overlap were then calculated. To make sure that the scale-free network calculated the pairwise Pearson correlation coefficients between each gene independently, a filtering threshold was applied to the pairwise correlation matrix, converting it to a neighborhood correlation matrix. Additionally, we calculated the eigenvector values of each module. After that, we performed hierarchical cluster analysis, computed the corresponding dissimilarities, and transformed the adjacency matrix into a topological overlap matrix. Finally, using gene significance values and module membership values, we assessed the relationships between gene modules and normal individuals as well as those with T2DM to identify key modules. The genes related to T2DM were identified at the intersection of DEGs and key modules revealed by WGCNA. The “limma,” “pheatmap,” “ggsci,” “ggplot2,” “dplyr,” “org.Hs.eg.db,” “patchwork,” “WGCNA,” “GSEABase,” “pheatmap,” “randomcoloR,” and “AnnoProbe” packages of the R software were used to identify the critical targets of T2DM.

### 2.7 Identification of key genes involved in PPI-induced T2DM

The Swiss TargetPrediction database (https://www.swisstargetprediction.ch/), Comparative Toxicogenomics database (CTD; https://ctdbase.org/) and Targetnet database (http://targetnet.scbdd.com/home/index/) were also employed to predict possible targets of PPIs (omeprazole, pantoprazole, lansoprazole, rabeprazole, esomeprazole, dexlansoprazole). The protein-protein interactions of PPI- and T2DM-related genes were investigated via the STRING website (https://string-db.org/). We used Cytoscape software to further optimize the protein-protein interaction networks, and the molecular complex detection (MCODE) algorithm was applied to screen the key clusters (Xie et al., 2022). The key genes involved in PPI-induced T2DM were identified at the intersection of T2DM-related genes, drug-related genes, and the key clusters. ROC curves of these key genes were plotted, and these targets were evaluated by computing the AUC of the ROC curve.

PPI-related genes and key clusters were analyzed by Gene Ontology (GO) and Kyoto Encyclopedia of Genes and Genomes (KEGG) for functional enrichment, respectively (Yu et al., 2012). Through GO analysis of these genes, three categories of cellular components, biological processes, and molecular functions were identified and used to examine the biological characteristics of PPI-related genes and key clusters. KEGG enrichment was utilized to identify the potential signaling pathways for PPI-related genes and key clusters. The “org.Hs.eg.db,” “clusterProfiler,” “enrichplot,” “ggplot2,” “ggnewscale,” “enrichplot,” “DOSE,” “ggpubr,” “stringr,” “ggsci,” “randomcoloR,” and “pathview” packages of the R software were used to perform GO analysis and KEGG enrichment.

## 3 Results

### 3.1 Correlation analysis

**FIGURE 2:**
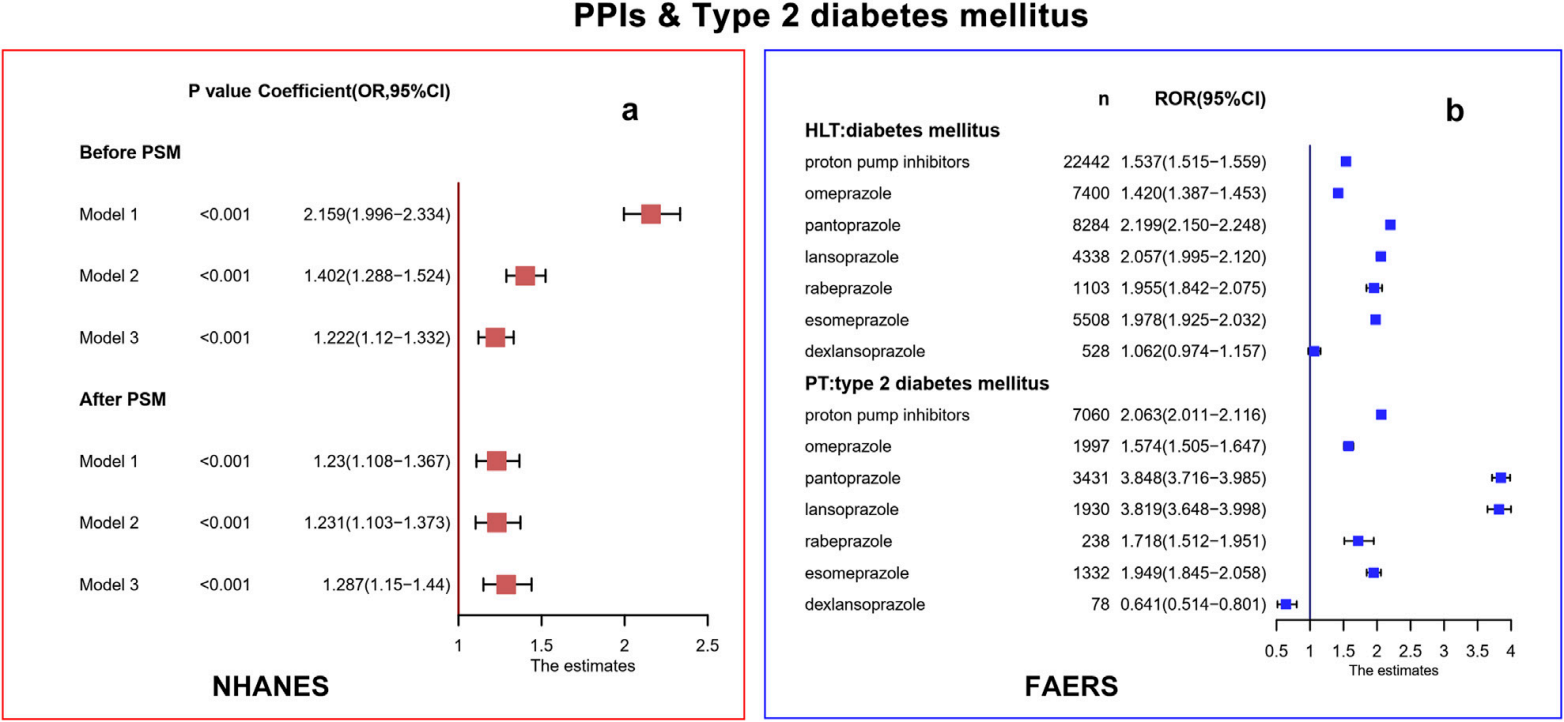
Correlation analysis in NHANES **(a)** and FAERS **(b)** database. PPIs, proton pump inhibitors; T2DM, type 2 diabetes mellitus; NHANES, National Health and Nutrition Examination Survey; FAERS, FDA Adverse Events Reporting System; CI, confidence interval; OR, odds ratio; PSM, propensity score matching; HLT, high-level terms; PT, preferred terms; ROR, report odds ratio.

In the NHANES database, we included 3,466 patients with PPI usage who received at least one episode of PPIs and 32,915 participants who did not receive any PPIs. Binomial logistic regression models indicated that the PPIs use group had a significantly higher risk of T2DM than the PPIs non-use group in model 1 (Coefficient: Odds Ratio [OR] = 2.159, 95% CI: 1.996–2.334, *p* < 0.001), model 2 (Coefficient: OR = 1.402, 95% CI: 1.288–1.524, *p* < 0.001) and model 3 (Coefficient: OR = 1.222, 95%CI: 1.120–1.332, *p* < 0.001) (Figure 2). Following PSM, 3,466 individuals were matched in the case and control groups. As shown in Supplementary Table S3, no significant differences were found between the two groups at baseline (*p* > 0.05). According to these three models, there was still a positive association between PPI usage and T2DM (Coefficient: model 1: OR = 1.230, 95%CI: 1.108–1.367, *p* < 0.001; model 2: OR = 1.231, 95%CI: 1.103–1.373, *p* < 0.001; model 3: OR = 1.287, 95%CI: 1.150–1.440, *p* < 0.001). Subgroup analyses showed omeprazole/esomeprazole/pantoprazole were significantly associated with the risk of developing T2DM in three models after PSM (Supplementary Table S4).

In the FAERS database, 7,060 PPI-related T2DM, 1,997 omeprazole-related T2DM, 3,431 pantoprazole-related T2DM, 1,930 lansoprazole-related T2DM, 238 rabeprazole-related T2DM, 1,332 esomeprazole-related T2DM, and 78 dexlansoprazole-related T2DM cases were contained. At the HLT level, the signal of diabetes mellitus in PPIs/omeprazole/pantoprazole/lansoprazole/rabeprazole was significant (lower limit of ROR was greater than 1). The T2DM signal at the PT level was associated with PPIs/omeprazole/pantoprazole/lansoprazole/rabeprazole at a significantly increased ROR (lower limit of ROR was greater than 1). Dexlansoprazole did not produce a signal of diabetes mellitus and T2DM (lower limit of ROR was lower than 1). The response signals obtained were completely consistent with the ROR results, and the PRR results are shown in Supplementary Table S5. After excluding patients who already had T2DM, hypertension, hyperlipidemia, and obesity, the signals of diabetes mellitus at the HLT level and T2DM at the PT level in PPIs/omeprazole/pantoprazole/lansoprazole/rabeprazole were also generated (Supplementary Table S6).

### 3.2 Prediction model construction

**FIGURE 3:**
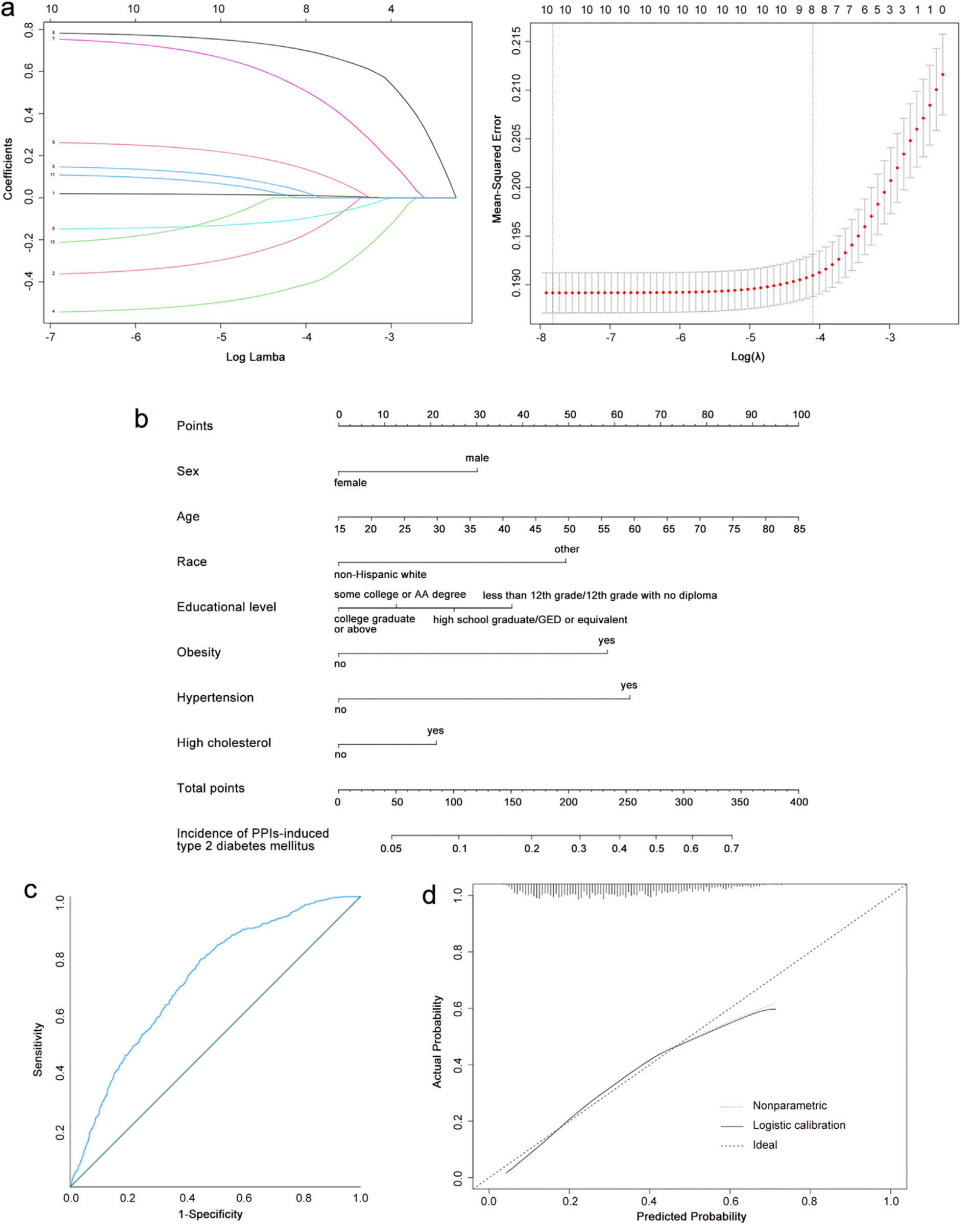
Construction of a PPI-induced T2DM risk prediction model. **(a)** Results of the LASSO regression. **(b)** Nomogram predicting T2DM risk among the people of PPI use. **(c)** Analysis of ROC curve for nomogram in the training group. **(d)** Calibration curves of the nomogram in the training group. PPIs, proton pump inhibitors; T2DM, type 2 diabetes mellitus; LASSO, least absolute shrinkage and selection operator; ROC, receiver operating characteristic; AUC, the area under the curve.

We included 3,467 patients to construct a PPI-induced T2DM prediction model. Because LASSO cannot handle unordered multi-categorical variables, we categorized the race of study participants into multiple dichotomous variables before performing LASSO regression. We selected the tuning parameter lambda in the LASSO model by 10-fold cross-validation based on the minimum criteria. According to the optimal lambda value (0.001018) for the LASSO regression, the results showed that eight variables with non-zero coefficients were selected as latent variables (Figure 3a). After performing a binomial logistic regression, the inclusion of seven optimal variables minimized the criterion value of Akaike information for the final model, which implies a better fit for the model.

Based on the final model, a nomogram was constructed to quantify the risk of PPI-induced T2DM (Figure 3b). The risk factors included sex, race, age, educational level, obesity, hypertension, and high cholesterol. Our nomogram expressed the predicted probability of PPI-induced T2DM risk on a scale of 0–350. For each predictor, a vertical line was drawn on the point axis and the corresponding points were noted. The points for each predictor were summed to find the total score corresponding to the predicted probability of PPI-induced T2DM risk at the bottom of the nomogram.

The C-index of the training group dataset was 0.712. For the testing group, the C-index was 0.740. The ROC curves for predicting the probability of T2DM risk in PPI users are shown in Figure 3c; Supplementary Figure S1A, with the AUC values of 0.790 (95% CI: 0.691, 0.734) in the training group and 0.740 (95% CI: 0.708, 0.771) in the testing group. The calibration curves of the training group and testing group demonstrated a high degree of concordance between the expected and actual results (Figure 3d; Supplementary Figure S1B). Our nomogram model performed well in predicting the risk of T2DM in the population using PPIs. According to the decision curve (Supplementary Figure S2), when the threshold probability was >5% for the patient and <53% for the clinicians, the nomogram(redline) would provide greater benefits than the treat-all-parents scheme(greyline) and treat-none scheme (black horizontal axis).

### 3.3 Identification of key genes involved

**FIGURE 4:**
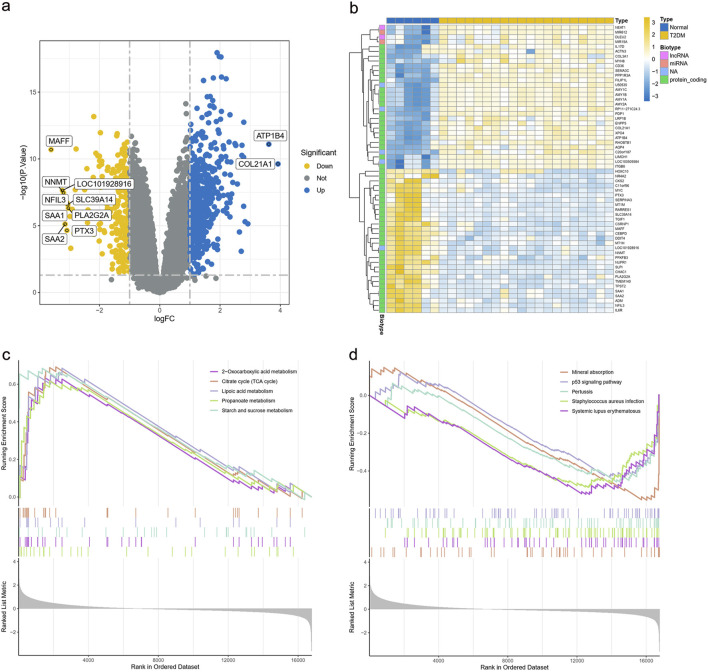
Expression of DEGs in the GSE7014 dataset. **(a)** Volcano plot of DEGs in the GSE7014 dataset. **(b)** Heatmap plot of DEGs in the GSE7014 dataset. **(c,d)** GSEA analysis of DEGs between the T2DM and normal groups DEGs, differentially expressed genes; T2DM, type 2 diabetes mellitus; GSEA, gene set enrichment analysis; KEGG, Kyoto Encyclopedia of Genes and Genomes.

We retrieved the gene expression data of 20 T2DM samples and 6 normal samples from the GEO database GSE7014 dataset. Before identifying genes that were differentially expressed between the control and T2DM groups, we first normalized the raw sequencing data (Supplementary Figure S3). Among these DEGs, 597 were upregulated and 239 were downregulated (Figure 4a). Sixty significant DEGs are displayed in the heatmap plot (Figure 4b). The DEGs pathway enrichment for the T2DM and normal groups was carried out by using GSEA analysis. The results of this analysis showed that 2-oxocarboxylic acid metabolism, the citrate cycle (tricarboxylic acid cycle), lipoic acid metabolism, propanoate metabolism, and starch and sucrose metabolism were enriched in the T2DM group (Figure 4c). The p53 signaling pathway, mineral absorption, pertussis, *staphylococcus aureus* infection, and systemic lupus erythematosus were inhibited (Figure 4d). These results imply that the development of T2DM may be significantly influenced by the metabolism of 2-oxocarboxylic acid.

**FIGURE 5:**
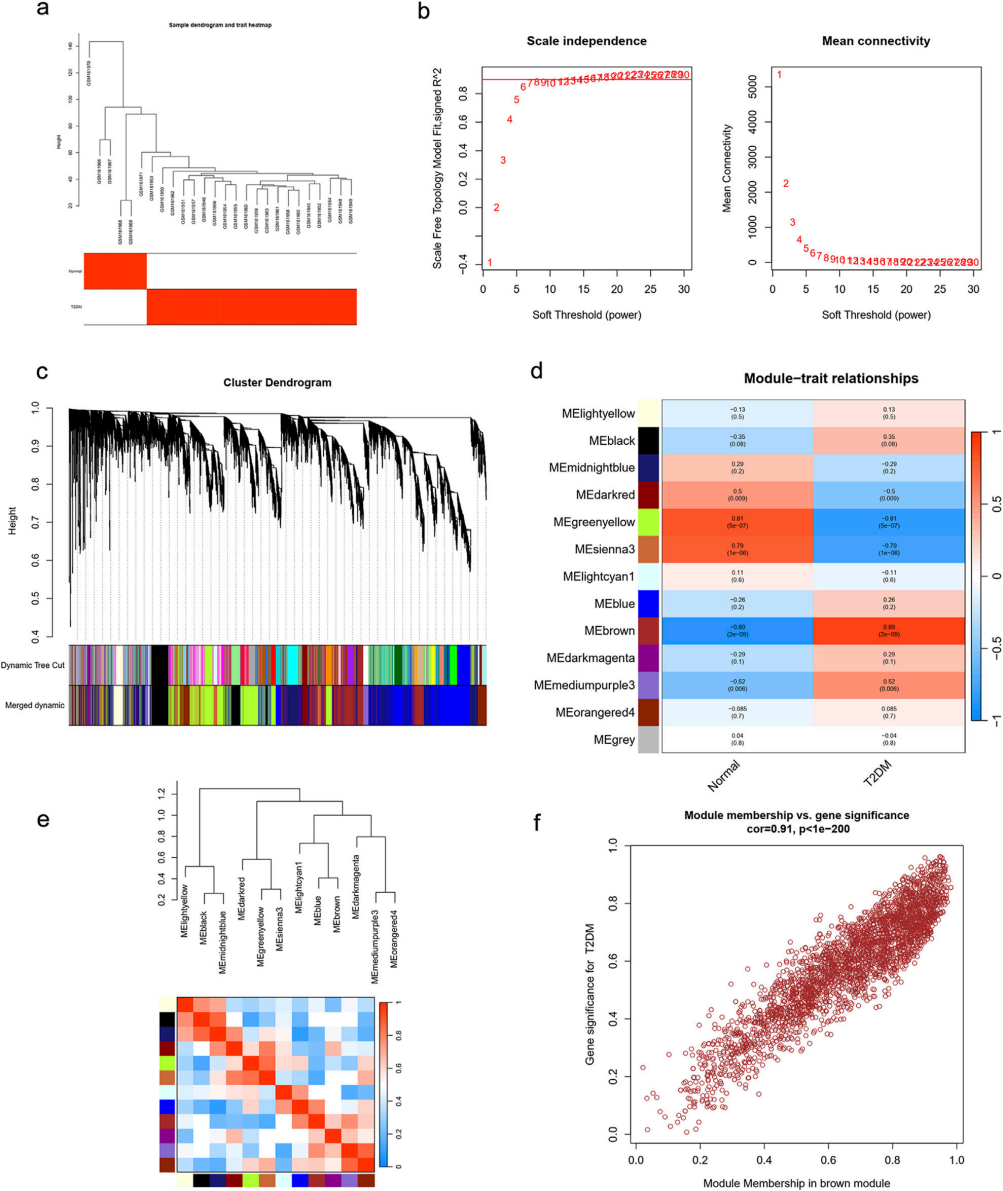
Enrichment levels in genomic WGCNA. **(a)** Sample dendrogram and trait heatmap. **(b)** Selection of soft thresholds. **(c)** Cluster dendrogram of WGCNA. **(d)** Correlations between gene modules and T2DM. **(e)** Correlation between modules. **(f)** Correlation between brown module memberships and gene significance. WGCNA, weighted correlation network analysis; T2DM, type 2 diabetes mellitus.

For the WGCNA approach, we used microarray data from the GSE7014 dataset. There were no notable outliers in the data, based on the outlier detection function (Figure 5a). A reasonable connection was shown by the scale-free index of 0.9 and the soft threshold power of 7 (Figure 5b). We created correlation matrices and topological overlaps between the genes in the data. By cutting dynamic trees and combining dynamic graphs, the co-expression network and clustering dendrograms were built (Figure 5c). Finally, 13 modules were categorized using the data clustering results (Figure 5d). We computed the association coefficient of each module with traits associated with T2DM. According to these results, the MEbrown module had the strongest correlation with T2DM (*p* = 2e-09, cor = 0.89). The heatmap of the correlation between these modules is displayed in Figure 5e. Based on the module membership and genetic significance scatter plots within the MEbrown module, the association was excellent (R = 0.91, *p* < 1e-200) (Figure 5f). As a result, the MEbrown module can be utilized as a T2DM phenotypic interpretation tool.

**FIGURE 6:**
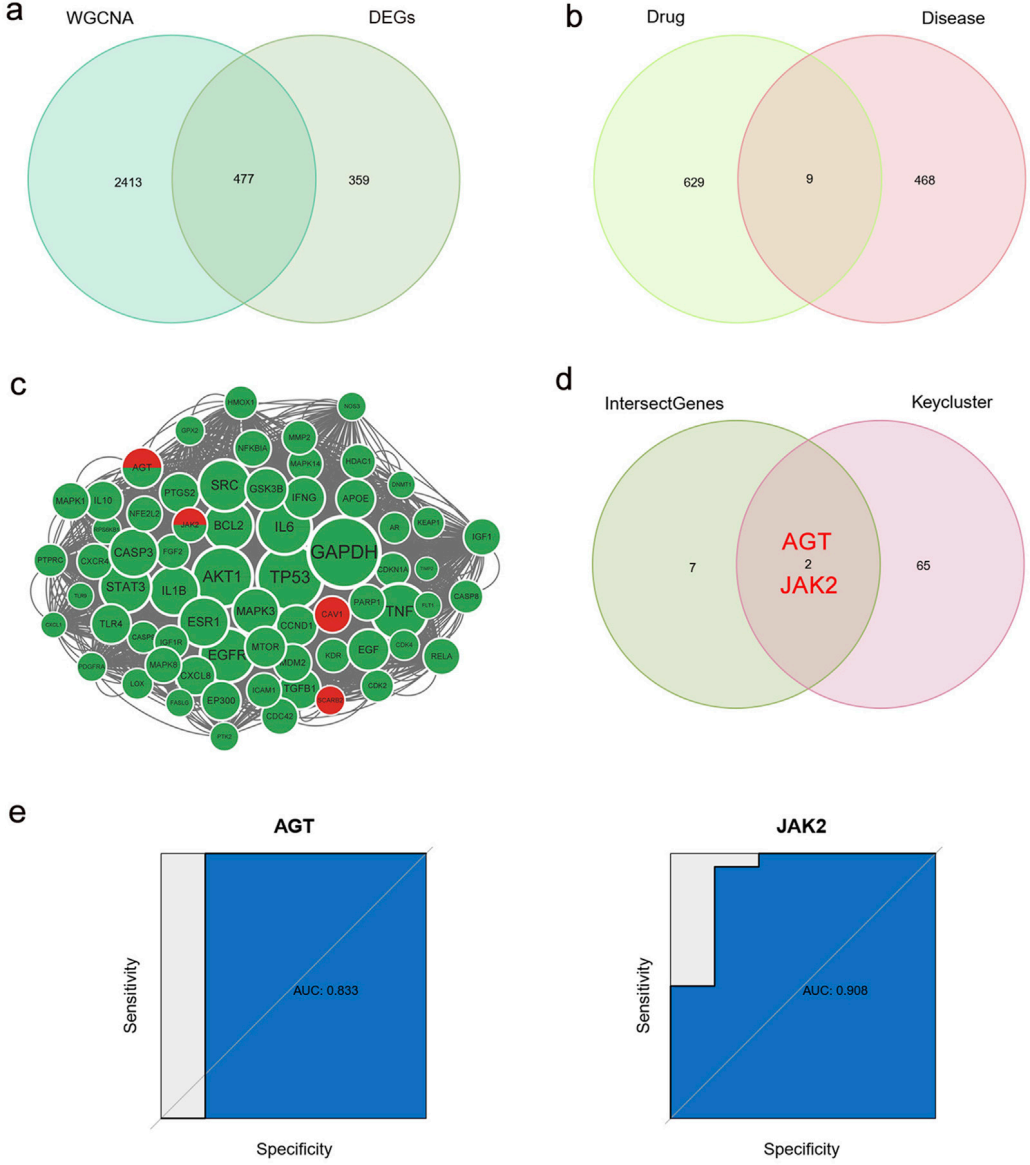
Identification of key genes. **(a)** Intersection of DEGs and WGCNA brown module genes, named T2DM-related genes. **(b)** Intersection of T2DM-related genes and PPI-related genes. **(c)** Cytoscape’s plugin code for all T2DM-related genes and PPI-related genes, named key cluster. Green: PPI-related genes; Red: T2DM-related genes; Green and red: both PPI-related genes and T2DM-related genes. **(d)** Intersection of key clusters, T2DM-related genes and PPI-related genes, named key genes. **(e)** ROC curve of two key genes. WGCNA, weighted correlation network analysis; DEGs, differentially expressed genes; T2DM, type 2 diabetes mellitus; PPIs, proton pump inhibitors; ROC, receiver operating characteristic.

We identified 477 T2DM-related genes by intersecting DEGs with MEbrown module genes (Figure 6a). Swiss TargetPrediction, CTD, and Targetnet databases yielded 205, 555, and 432 PPI-related targets, respectively (Supplementary Table S7). Nine genes were identified in the intersection between the T2DM- and PPI-related genes (Figure 6b). The STRING online database was used to build a protein-protein interaction network for all T2DM- and PPI-related genes. Sixty-seven essential subpopulation genes, called key clusters, were identified by using the MCODE algorithm (Figure 6c). We found two key genes, AGT and JAK2, by a comparison of key clusters and intersecting genes from the T2DM- and PPI-related genes (Figure 6d; Supplementary Figure S4). Based on the ROC curves, these two genes were robustly associated with T2DM (AUC of ROC curve >0.8) (Figure 6e).

The results of GO and KEGG analyses of PPI-related genes and key clusters are shown in Supplementary Figures S5–S8. The biological process category in GO was co-enriched in response to oxidative stress and the cellular response to chemical stress. The GO cellular component category was co-enriched in membrane raft, membrane microdomain, vesicle lumen, cytoplasmic vesicle lumen, focal adhesion, and caveolae. The molecular function section was co-enriched in protein tyrosine kinase activity (Supplementary Figures S5, S7). The KEGG pathway primarily showed an enrichment of PPI-related genes while key clusters were co-enriched in lipids and atherosclerosis, the AGE-RAGE signaling pathway in diabetic complications, endocrine resistance, and other related pathways (Supplementary Figures S6, S8).

## 4 Discussion

Previously, the association between PPI use and T2DM risk has been controversial. Our study is the first comprehensive study to explore the relationship between PPI use and T2DM risk, based on large-scale datasets from the NHANES and FAERS databases. These databases undergo rigorous quality control and standardization processes to ensure the accuracy and consistency of their data. In the NHANES database, regardless of whether PPIs were merely included or used to adjust for disease or all covariates, the binomial regression models found a positive correlation between PPI use and T2DM risk, and these results were not reversed after PSM. Additionally, the signal of T2DM in PPI use was also significantly detected by ROR and PRR methods in the dataset derived from the FAERS database. Even after excluding patients who already had T2DM, hypertension, hyperlipidemia, and obesity, the results were not reversed. We developed and validated a nomogram based on the 1999–2017 dataset from the NHANES database to predict the probability of PPI-induced T2DM risk. Our nomogram included seven variables (sex, race, age, educational level, obesity, hypertension, and high cholesterol), each of which can be easily acquired from PPI treatment. Finally, we combined network pharmacology, difference analysis, WGCNA, GSEA, GO, and KEGG analysis to identify the genes potentially associated with PPI-induced T2DM risk. By intersecting the key cluster and the intersection of PPI- and T2DM-related genes, we were able to identify two genes, AGT and JAK2, which may play critical roles in PPI-induced T2DM development.

In line with the findings of most studies, our study also demonstrated that PPI usage may be capable of increasing the risk of developing T2DM. In a prospective population-based cohort of 14,926 individuals (Rotterdam Study), researchers found that PPI usage was linked to a higher risk of incident T2DM (Hazard Ratio [HR] = 1.69, 95% CI: 1.36–2.10). The effect was dose-dependently associated with the highest risk (HR = 1.88, 95% CI: 1.29–2.75) (Czarniak et al., 2022). Another high-impact prospective study of 20,4689 participants also showed that regular PPI users had a 24% greater risk of diabetes than non-users (HR = 1.24, 95% CI: 1.17–1.31), and the risk of diabetes likewise rose with the length of PPI use (Yuan et al., 2021). Loosen et al. used the Disease Analyser database of 26,744 patients diagnosed with T2DM to re-confirm Yuan et al.'s findings, offering strong proof of a link between frequent PPI usage and the likelihood of developing T2DM (Loosen et al., 2022). Chenchula et al. pooled 12 studies with a total patient cohort of 1,264,816 persons to reveal that PPI use has a significant association with the risk of T2DM incidence (*p* < 0.001) (Chenchula et al., 2024). In our present study, the analysis of two large-sample databases also indicated a correlation between PPI use and the risk of developing T2DM. Conversely, some studies have revealed no relationship between PPI therapy and a reduction in non-glycosylated HbA1c levels and have even observed a reduction in the risk of diabetes (Lin et al., 2016; Han et al., 2015; Trang et al., 2021). While, the conclusions reached by these studies were either obtained from retrospective reviews, or included a small number of participants, and the evidence-based support for these studies is relatively low. We mutually validated our findings through two large-sample real-world databases and took into account potential confounders to enhance the credibility of our study. Therefore, our findings showed that PPI therapy could increase the risk of T2DM.

By referring to published literature, there was no study on the construction of a prediction model for PPI-induced T2DM risk. Over the past 3 decades, we have witnessed an almost continuous growth in the use of PPIs on a global scale. For example, in 2010, omeprazole was the number one drug sold in Spain, accounting for 5.5% of total drug package invoices (Lanas, 2016). Similarly, in the period from 2022–2023, 73 million PPI prescriptions were dispensed by the National Health Service in the United Kingdom, at a cost of approximately £190 million, which is equivalent to 1.8% of the total cost of primary care prescriptions and 6% of all prescriptions written (Jenkins and Modolell, 2023). The global impact of diabetes, including T2DM, is severe, costing more than $760 billion and representing 10% of the annual health expenditures for adults, making it an urgent global concern (Cavan et al., 2017). Therefore, establishing a simple and practical prediction model appears particularly essential to help clinicians quickly and accurately identify patients potentially at T2DM risk from the use of PPIs. Applying LASSO regression analysis and developing a nomogram model helped us in extracting the optimal variables most closely associated with the incidence of T2DM in PPI users, thus paving the way for identifying a high-risk population among PPI users based on the selection of patient characteristics derived from clinical practice. Moreover, each of these characteristics of our nomogram is readily available from PPI users, and our nomogram is plausible that has shown good performance in the cohort.

By identifying key genes, we were able to explore the possible mechanisms by which PPIs may cause T2DM. The protein encoded by the AGT gene, pre-angiotensinogen or angiotensinogen precursor, is cleaved to generate the physiologically active enzyme angiotensin II. Angiotensin II is the primary active product of the Renin-Angiotensin system. By binding to angiotensin II receptor type 1 receptors, angiotensin II activates downstream MAPK and JNK signaling pathways, leading to the inhibition of the insulin signaling pathway and subsequently causing insulin resistance (Gutierrez-Rodelo et al., 2022). Joyce-Tan et al. found that the genetics of the AGT gene were associated with an increased risk of T2DM (OR = 1.92, 95% CI:1.15–3.20, permuted *p* = 0.012) through genetic testing of 557 Malay participants (Joyce-Tan et al., 2016). Hirode et al. analyzed a large-scale transcriptome database of rat liver and showed that omeprazole affected the expression of AGT mRNA (Hirode et al., 2009). The expression of angiotensin II occurs in the gastric mucosa, and PPIs can reduce gastric acid secretion, which may indirectly affect the local renin-angiotensin system in the gastric mucosa (Duarte et al., 2024). From this observation, we hypothesized that PPIs may contribute to the increased risk of T2DM by modulating angiotensin expression via the AGT gene. Another key gene encodes non-receptor tyrosine kinases that are involved in a variety of cell signaling pathways, with described to be associated with aging, inflammation, hematopoiesis, and malignant transformation (Perner et al., 2019). Zhang et al. recruited 599 T2DM patients for genetic testing and found that the JAK2 gene may be correlated with T2DM in the Chinese population (Zhang et al., 2022). The JAK2/STAT3 signaling pathway is involved in the development of insulin resistance in patients with T2DM, and its abnormal activation may damage pancreatic beta cells to reduce insulin synthesis (Mashili et al., 2013; Bharadwaj et al., 2020). Enhanced JAK2 expression promotes the progression of diabetic nephropathy (Cao et al., 2023). Nikzamir et al. used network analysis to evaluate the genes deregulated after long-term consumption of omeprazole. The critically deregulated genes we finally identified were JAK2, PTK2, and NRG1 (Nikzamir et al., 2020). PPIs can modulate the activity of macrophages and dendritic cells in the gastric mucosa, thus influencing the activation of the JAK2/STAT3 signaling pathway (Nie and Yuan, 2020). In the GO analysis undertaken by our study, molecular functional selection was co-enriched in protein tyrosine kinase activity. The KEGG pathway analysis results showed a co-enrichment for endocrine resistance and the AGE-RAGE signaling pathway in diabetic complications. Therefore, the JAK2 signaling pathway may be involved in the development of T2DM caused by PPI use.

It should be noted that our study had some limitations. First, in the NHANES database, in order to expand the sample size, we accessed the Prescription Medication Section to include patients taking PPIs without considering the effects of other medications. So there was no estimate of the effects of combination medications. T2DM is a multifactorial disease involving multiple genetic and environmental factors, but these factors could not be included in our present analysis because they were not explicitly documented in the NHANES database. A larger cohort study is warranted to further explore the seven results incorporated into our predictive model. Second, the FAERS database is a spontaneous reporting system that is prone to risks of reporting biases, including under-reporting, misreporting, and selective reporting. Although our study employed both the ROR and PRR methods for signal detection to ensure the reliability of the results, the influence of reporting biases cannot be denied, which is an inherent limitation of the FAERS system (Honma et al., 2024). Meanwhile, the NHANES and FAERS data remain cross-sectional, represent only statistical associations and do not imply causation, and longitudinal or prospective studies are warranted to better clarify the direction of the association (Raschi et al., 2021). Third, our study only used bioinformatics technology to conduct a preliminary exploration of the possible gene targets and mechanisms involved in the development of T2DM induced by PPIs. Further experiments are required to validate the ability of PPIs to bind and inhibit key gene targets (e.g., affinity assays, gene expression, *in vitro* inhibition assays, and direct mutation studies). Additionally, cellular or animal studies should be used to further validate the true impact of the JAK2 signaling pathway and angiotensin II in the development of T2DM driven by use of PPIs.

## 5 Conclusion

This study combined the NHANES and FAERS databases to explore the relationship between PPI use and T2DM risk. Our analysis of datasets obtained from these two large-scale databases indicated a positive relationship between PPI use and the risk of developing T2DM. PPIs are in widespread use worldwide, and T2DM is also a major public health problem globally. Clinical workers should be wary of T2DM induced by PPIs. Then, we applied LASSO regression analysis and developed a nomogram model based on data from the NHANES database to predict the probability of PPI-induced T2DM risk. Our model can facilitate clinicians in identifying high-risk groups based on patient characteristics and take timely preventive and avoidance measures in clinical practice. Finally, we combined a variety of bioinformatics techniques to identify the potential genes (AGT and JAK2) that may be involved in PPI-induced T2DM risk. We also carried out a preliminary exploration of the possible mechanisms of PPI-induced T2DM development. However, more cohort studies and experiments are needed to verify our model and conclusions.

## Data Availability

The original contributions presented in the study are included in the article/Supplementary Material, further inquiries can be directed to the corresponding author.
