# Supplementary material for: Proton pump inhibitors use and risk of type 2 diabetes mellitus: correlation analysis, prediction model construction, and key genes identification
Source: Front Pharmacol. 2025 Apr 29;16:1580090. doi: 10.3389/fphar.2025.1580090 (PMC12069289; doi:10.3389/fphar.2025.1580090)
Supplement: Supplementary file 1 [file DataSheet1.pdf]

Supplementary Table 1. The 2×2 table for disproportionality analysis

	Drug(s) of interest	Other drugs	Total
Adverse events of interest	a	b	a+b
Other adverse events	c	d	c+d
Total	a+c	b+d	a+b+c+d

Supplementary Table 2. Formulas for the 2 algorithms in disproportionality analysis

Model	Formula	Thresholds
ROR	ROR=(a/b)/(c/d)	a≥3
		the lower limit of the 95% CI of the ROR >1
PRR	PRR=[a/(a+c)]/[b/(b+d)]	a≥3
		the lower limit of the 95% CI of the PRR >1

ROR, reporting odds ratio; PRR, proportional reporting ratio; CI, confidence interval

Supplementary Table 3. Characteristics of NHANES participants

	Before PSM		After PSM	
Variable	PPIs non-user	PPIs user	PPIs non-user	PPIs user
Age	50.066±0.097	61.909± 0.240	62.369±0.239	61.902±0.240
Gender				
Male	16710(51.87%)	1697(48.95%)	1762(50.84%)	1697(48.96%)
Female	15506(48.13%)	1770(51.05%)	1704(49.16%)	1769(51.04%)
Race				
Mexican American and other Hispanic	7307(22.68%)	604(17.42%)	639(18.44%)	604(17.43%)
Non-Hispanic White	14951(46.41%)	2051(59.16%)	1977(57.04%)	2050(59.15%)
Non-Hispanic Black and Other Race	9958(30.91%)	812(23.42%)	850(24.52%)	812(23.43%)
Hypertension				
Yes	11586(35.96%)	2182(62.94%)	2170(62.61%)	2181(62.93%)
No	20630(64.04%)	1285(37.06%)	1296(37.39%)	1285(37.07%)
Obesity				
Yes	12242(38.00%)	1648(47.53%)	1589(45.85%)	1647(47.52%)
No	19974(62.00%)	1819(52.47%)	1877(54.15%)	1819(52.48%)
Smoking status				
Never	16435(51.02%)	1362(39.28%)	1390(40.10%)	1362(39.30%)
Former	9075(28.17%)	1442(41.59%)	1414(40.80%)	1441(41.58%)
Current	6706(20.82%)	663(19.12%)	662(19.10%)	663(19.13%)
Educational level				
Less than 12th grade (Includes 12th grade with no diploma)	6666(20.69%)	889(25.64%)	884(25.50%)	889(25.65%)
High school graduate/GED	7301(22.66%)	890(25.67%)	877(25.30%)	889(25.65%)
Some college or AA degree	9977(30.97%)	1019(29.39%)	1030(29.72%)	1019(29.40%)
College graduate or above	8272(25.68%)	669(19.30%)	675(19.47%)	669(19.30%)
Alcohol status				
Yes	4151(12.88%)	335(9.66%)	292(8.42%)	335(9.67%)
No	28065(87.12%)	3132(90.34%)	3174(91.58%)	3131(90.33%)
High cholesterol				
Yes	11707(36.34%)	2004(57.80%)	1954(56.38%)	2003(57.79%)
No	20509(63.66%)	1463(42.20%)	1512(43.62%)	1463(42.21%)

NHANES, National Health and Nutrition Examination Survey; PSM, propensity score matching; PPI, PPIs, proton pump inhibitors

Supplementary Table 4. Correlation analysis between omeprazole/esomeprazole/pantoprazole and T2DM in NHANES database after PSM.

	P value	Cofficient (OR, 95%CI)
Omeprazole (n=1675)		
Model 1	<0.001	1.374(1.183-1.597)
Model 2	<0.001	1.400(1.197-1.638)
Model 3	<0.001	1.434(1.220-1.686)
Esomeprazole (n=614)		
Model 1	0.043	1.292(1.008-1.658)
Model 2	0.033	1.324(1.024-1.713)
Model 3	0.024	1.353(1.041-1.762)
Pantoprazole (n=576)		
Model 1	0.046	1.307(1.006-1.701)
Model 2	0.027	1.359(1.035-1.787)
Model 3	0.028	1.372(1.035-1.821)

T2DM, type 2 diabetes mellitus; NHANES, National Health and Nutrition Examination Survey; OR, odds ratio; CI, confidence interval; PSM, propensity score matching

Supplementary Table 5. PRR and 95% CI of PPIs associated diabetes mellitus reported in FAERS.

	n	PRR (95% CI)
HLT: diabetes mellitus (incl subtypes)		
proton pump inhibitors	22442	1.530(1.509-1.552)
omeprazole	7400	1.415(1.383-1.448)
pantoprazole	8284	2.178(2.130-2.226)
lansoprazole	4338	2.039(1.979-2.101)
rabeprazole	1103	1.940(1.829-2.057)
esomeprazole	5508	1.963(1.911-2.016)
dexlansoprazole	528	1.061(0.975-1.155)
PT: type 2 diabetes mellitus		
proton pump inhibitors	7060	2.059(2.007-2.112)
omeprazole	1997	1.573(1.504-1.645)
pantoprazole	3431	3.828(3.697-3.963)
lansoprazole	1930	3.798(3.629-3.975)
rabeprazole	238	1.715(1.510-1.948)
esomeprazole	1332	1.945(1.842-2.054)
dexlansoprazole	78	0.642(0.514-0.801)

FAERS, FDA Adverse Events Reporting System; PPIs, proton pump inhibitors; PRR, Proportional Reporting Ratio; CI, confidence interval; HLT, high-level terms; PT, preferred terms

Supplementary Table 6. ROR, PRR and 95% CI of PPIs associated diabetes mellitus in model 2*.

	n	ROR	PRR
	HLT:diabetes mellitus (incl subtypes)		
proton pump inhibitors	22442	1.954(1.924-1.985)	1.941(1.912-1.971)
omeprazole	7400	1.445(1.408-1.483)	1.44(1.404-1.478)
pantoprazole	8284	2.367(2.31-2.424)	2.343(2.289-2.4)
lansoprazole	4338	2.188(2.117-2.262)	2.169(2.1-2.241)
rabeprazole	1103	2.055(1.924-2.195)	2.039(1.911-2.176)
esomeprazole	5508	2.094(2.034-2.157)	2.078(2.018-2.139)
dexlansoprazole	528	1.091(0.994-1.198)	1.09(0.994-1.196)
	PT: type 2 diabetes mellitus		
proton pump inhibitors	7060	2.556(2.487-2.628)	2.549(2.481-2.62)
omeprazole	1997	1.486(1.413-1.563)	1.485(1.412-1.561)
pantoprazole	3431	4.148(3.998-4.305)	4.125(3.976-4.28)
lansoprazole	1930	4.155(3.959-4.36)	4.131(3.938-4.334)
rabeprazole	238	1.828(1.594-2.096)	1.825(1.592-2.092)
esomeprazole	1332	1.966(1.853-2.087)	1.963(1.85-2.082)
dexlansoprazole	78	0.613(0.479-0.783)	0.613(0.48-0.783)

*Model 2: patients were excluded who already had T2DM, hypertension, hyperlipidemia, and obesity before therapy

PPIs, proton pump inhibitors; ROR, reporting odds ratio; PRR, Proportional Reporting Ratio; CI, confidence interval; HLT, high-level terms; PT, preferred terms

Supplementary Table 7. Related targets of PPIs from different databases

	SwissTargetPrediction database	CTD	Targetnet databases
omeprazole	22	431	75
pantoprazole	20	27	61
lansoprazole	19	78	71
rabeprazole	21	18	79
esomeprazole	22	1	75
dexlansoprazole	101	0	71
total	205	555	432

CTD, Comparative Toxicogenomics database

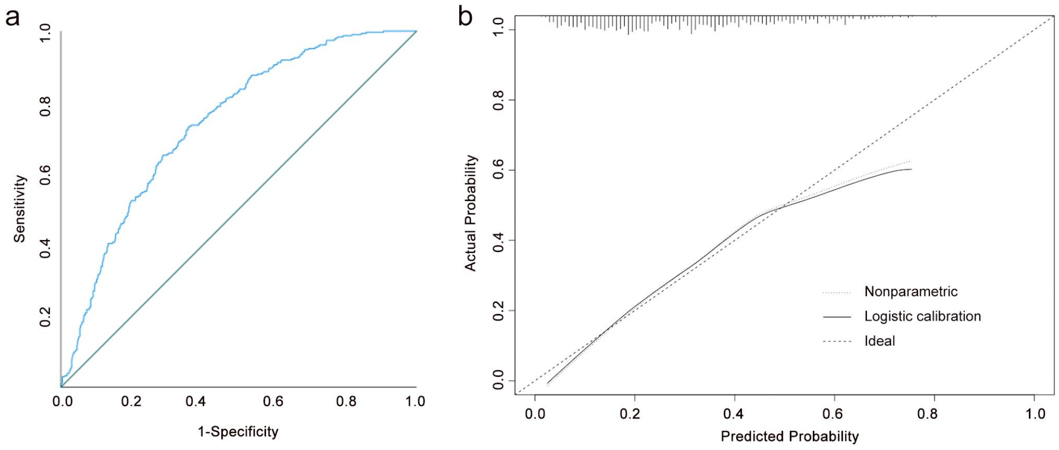

Supplementary Figure S1. Analysis of ROC curve(a) and calibration curves(b) of the nomogram in the testing group.

ROC, receiver operating characteristic

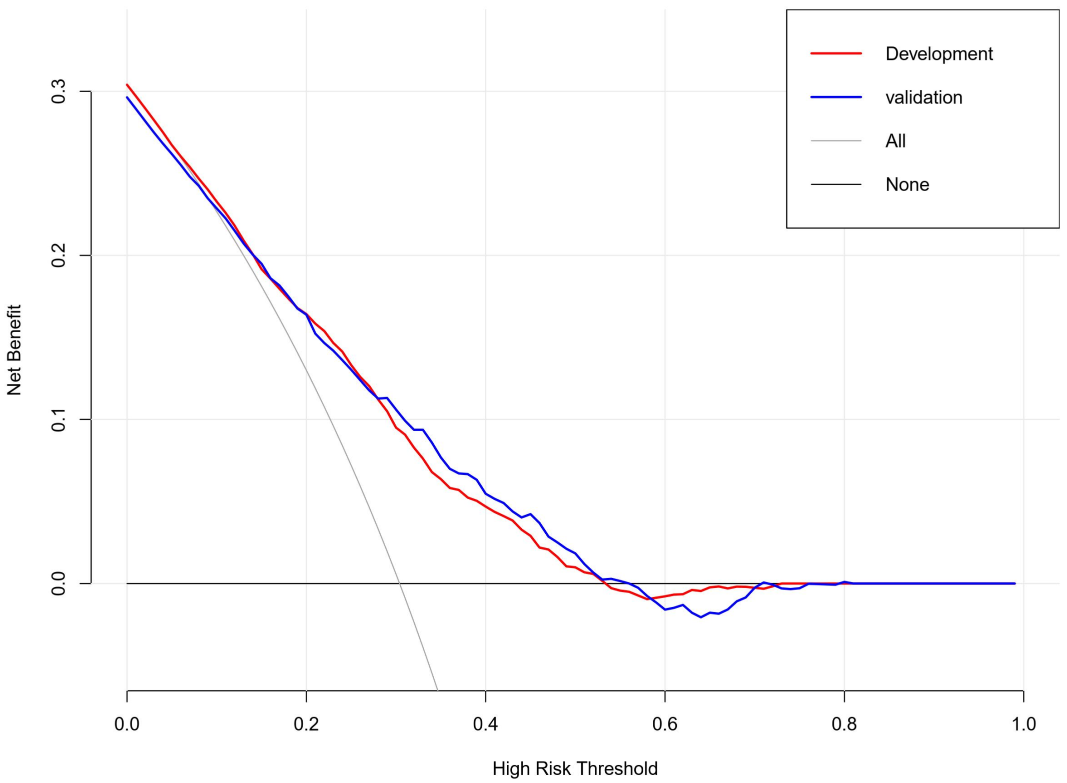

Supplementary Figure S2. Decision curve assessment for the nomogram.

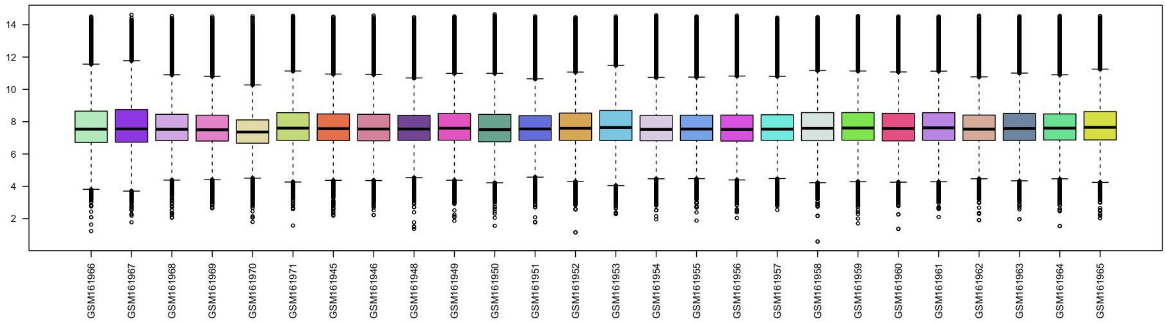

Supplementary Figure S3. normalized raw sequencing data of GSE7014

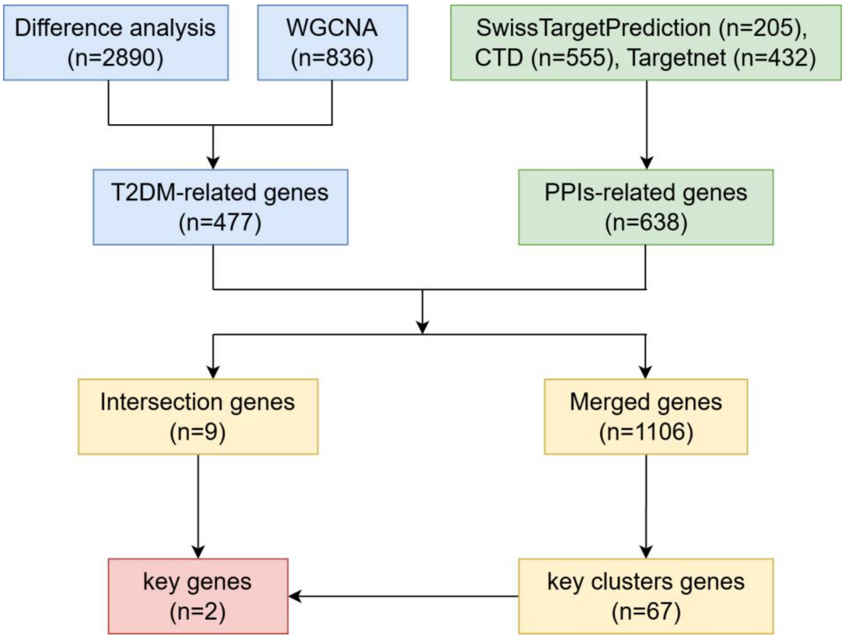

Supplementary Figure S4. Flowchart of key genes screening

WGCNA, weighted correlation network analysis; PPIs, proton pump inhibitors; T2DM, type 2 diabetes mellitus; CTD, Comparative Toxicogenomics database

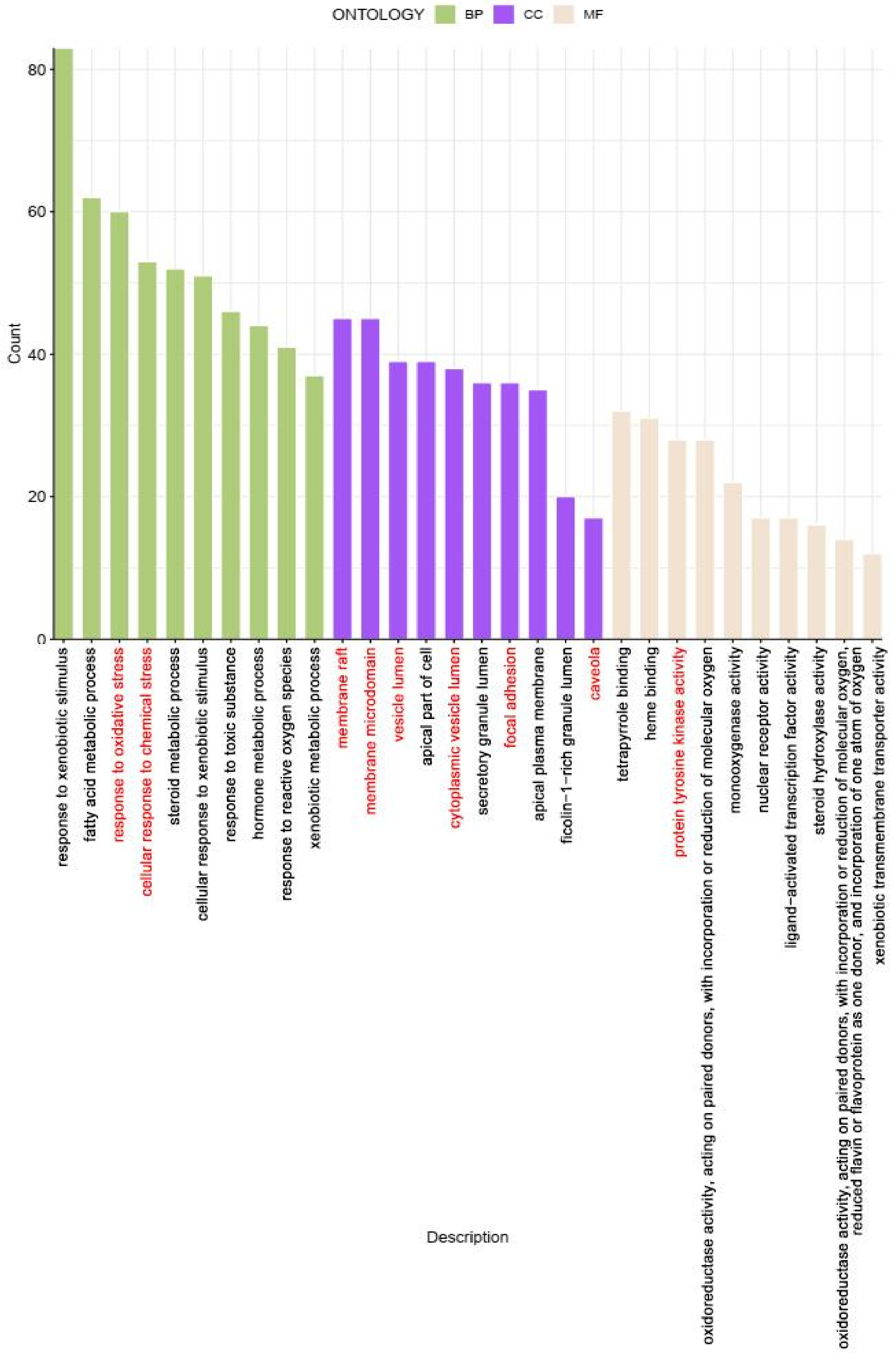

Supplementary Figure S5. GO analysis of the PPIs-related genes.

PPIs, proton pump inhibitors; GO, Gene Ontology; CC, cellular components; BP, biological processes; MF, and molecular functions.

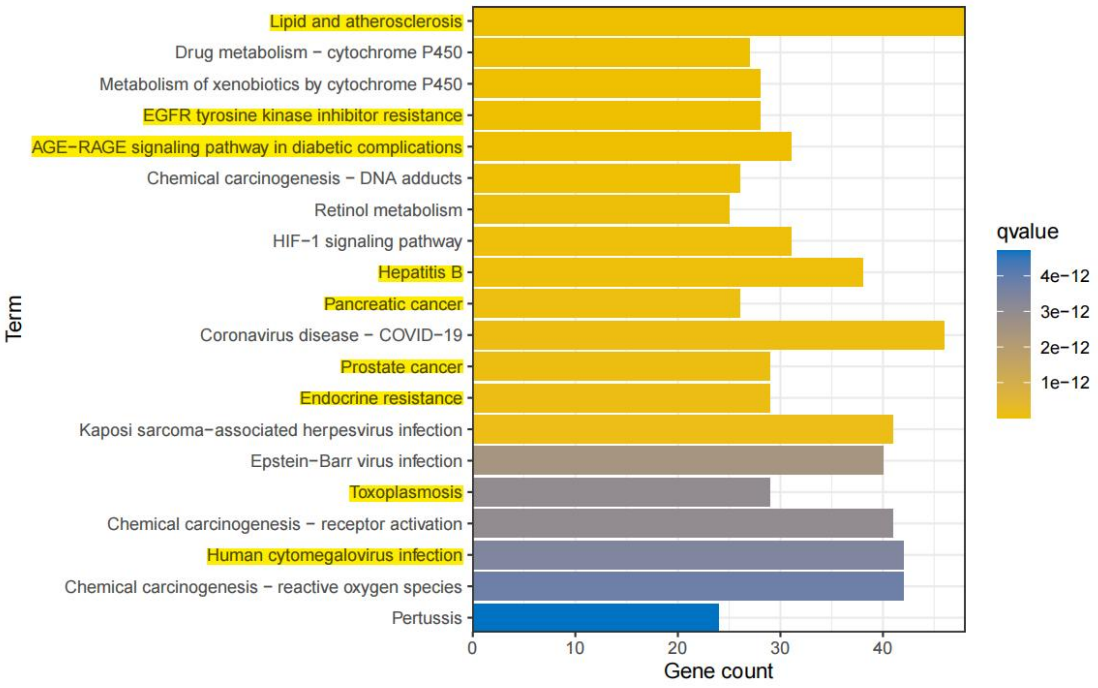

Supplementary Figure S6. KEGG analysis of PPIs-related genes.

PPIs, proton pump inhibitors; KEGG, Kyoto Encyclopedia of Genes and Genomes.

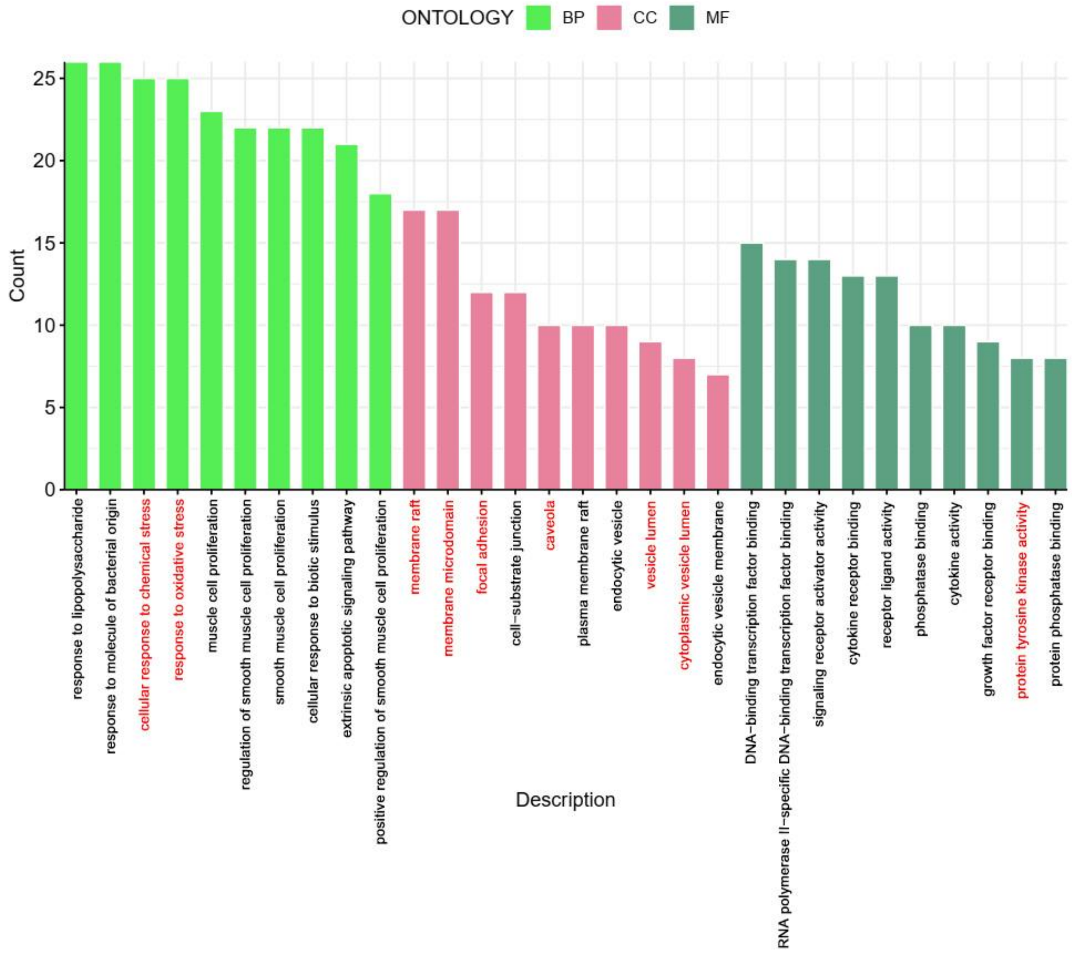

Supplementary Figure S7. GO analysis of the key cluster.

GO, Gene Ontology; CC, cellular components; BP, biological processes; MF, and molecular functions.

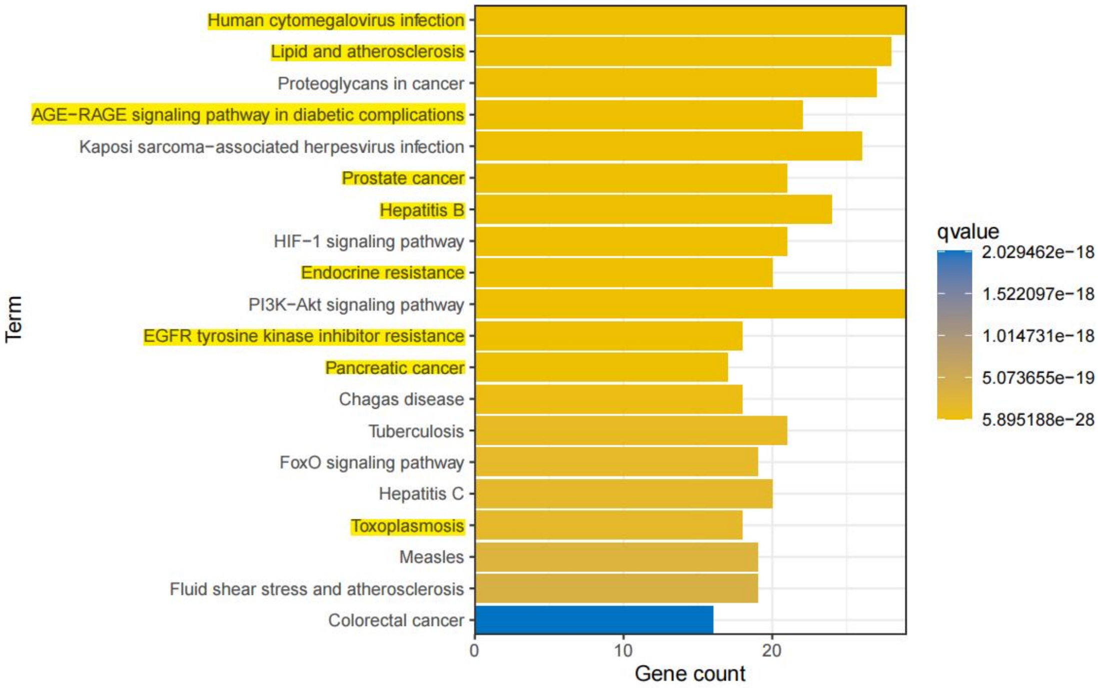

Supplementary Figure S8. KEGG analysis of key cluster.

KEGG, Kyoto Encyclopedia of Genes and Genomes.
